# Role of BCR-ABL1 isoforms on the prognosis of Philadelphia chromosome positive acute lymphoblastic leukemia in the tyrosine kinase inhibitor era: A meta-analysis

**DOI:** 10.1371/journal.pone.0243657

**Published:** 2020-12-18

**Authors:** Wanhua Zhang, Pu Kuang, Ting Liu

**Affiliations:** Department of Hematology, West China Hospital, Sichuan University, Chengdu, Sichuan Province, China; The University of Adelaide, AUSTRALIA

## Abstract

BCR-ABL1 fusion gene is the driver mutation of Philadelphia chromosome positive acute lymphoblastic leukemia (Ph+ ALL). Although the prognostic value of BCR-ABL1 isoforms in Ph+ ALL patients has been investigated in numerous studies in the tyrosine kinase inhibitor (TKI) era, the results were still conflicting. Hence we performed herein the meta-analysis to comprehensively assess the impact of BCR-ABL1 isoforms on the clinical outcomes of Ph+ ALL patients. Systematic literature review was conducted in PubMed, Embase, and Cochrane databases with the data access date up to June 15, 2020. Pooled hazard ratios (HRs) with 95% confidence intervals (CIs) were calculated with fixed-effects or random-effects models. Furthermore, subgroup analyses were performed to assess the robustness of the associations. Nine studies with a total number of 1582 patients were eligible for this meta-analysis. Combined HRs suggested that p210 was slightly associated with inferior event-free survival (EFS) (HR = 1.34, 95% CI 1.05–1.72). The overall survival (OS) was not significantly affected (HR = 1.15, 95% CI 0.92–1.45). In subgroup analyses, the HRs showed a trend toward adverse impact of p210 on clinical outcomes. However, the confidence intervals were not crossing the null value only in a minority of subgroups including Caucasian studies, first-generation TKI treated cohort and transplant cohort. Our findings suggested that p210 might pose a mild adverse impact on the EFS of Ph+ ALL patients. This effect might be compromised by the use of second- or third-generation TKIs. Further studies are needed to verify our conclusions.

## Introduction

Philadelphia chromosome positive acute lymphoblastic leukemia (Ph+ ALL) is one of the most commonly encountered genetic subtypes of adult ALL, with the occurrence of about 25% in ALL patients. And its frequency increases with age, reaching 40–45% in patients above 45 years [1, 2]. Historically, Ph+ ALL was considered as the most dismal subtype of ALL, with long-term survival of less than 20%, and allogeneic hematopoietic stem cell transplantation (allo-HSCT) was the mainstay of post-remission therapy [3–5]. The advent of tyrosine kinase inhibitors (TKIs) targeting the BCR-ABL1 oncoprotein has revolutionized the treatment response and long-term survival of Ph+ ALL patients, making the outcome equivalent to or better than that of Ph- ALL patients [6–10]. Despite the dramatic outcome improvement in this group of patients, the long-term disease-free survival of 40–50% is still less than satisfactory. Relapse and drug resistance remains a clinical challenge. Recognition of potential risk factors for relapse and mortality could help inform clinical decisions for this heterogeneous disease entity. As allo-HSCT might be dispensable in some patients, and some patients might need intensified treatment regimens including allo-HSCT.

The Philadelphia (Ph) chromosome is the der(22) product of the reciprocal translocation between 9q34 and 22q11.2. This translocation joins almost the entire coding region of the ABL1 (Abelson murine leukemia viral oncogene homolog 1) tyrosine kinase gene on chromosome 9 to the breakpoint cluster region (BCR) gene on chromosome 22, generating the BCR-ABL1 fusion gene [11]. There are two main breakpoint regions within the BCR gene, namely major or minor breakpoint cluster region (M-BCR or m-BCR). M-BCR lies between exons 12 and 16, producing the larger p210 oncoprotein, while the m-BCR yields a smaller p190 product that retains only the first exon of BCR. A rare breakpoint between exons 19 and 20 has also been described, giving rise to the p230 fusion protein [12]. Most childhood Ph+ ALL patients carry p190, whereas in adult Ph+ ALL, p190 and p210 are seen in 50–70% and 30–50% of the patients, respectively [13]. In transforming assays and transgenic mouse models, p190 exhibited stronger transforming potency than p210 [14–16]. The impact of BCR-ABL1 isoforms on the prognosis of Ph+ ALL patients has been investigated in numerous studies. However, the results remain conflicting. Hence we performed herein a systemic literature review and meta-analysis to quantitatively clarify its potential value as a prognostic biomarker.

## Materials and methods

### Literature search and study selection

This meta-analysis was performed and reported according to the PRISMA statement [17]. A PRISMA checklist (S1 Checklist) was used to ensure standardized reporting. The review protocol has been registered in the PROSPERO international prospective register of systematic reviews (registration number: CRD42020206891).

A systematic literature search was conducted in the PubMed, Embase and Cochrane databases with the data access date up to June 15, 2020 with free-style words and Medical Subjects Headings (MeSH): (("Philadelphia Chromosome"[Mesh] OR BCR-ABL1) AND "Precursor Cell Lymphoblastic Leukemia-Lymphoma"[Mesh]) OR Ph positive acute lymphoblastic leukemia OR BCR-ABL1 positive acute lymphoblastic leukemia. The search strategy for Pubmed was provided in S1 File. References of the included studies in this meta-analysis and relevant reviews were also screened for potentially eligible studies.

Two authors independently assessed the eligibility of the studies according to the following criteria: (1) randomized controlled trials (RCT) or cohort studies that compared the clinical outcomes of Ph+ ALL patients with p190 or p210 BCR-ABL1 isoforms; (2) TKIs were included in the treatment regimens; (3) the study outcomes were time-dependent endpoints, such as disease-free survival (DFS)/relapse-free survival (RFS)/event-free survival (EFS) and overall survival (OS); (4) hazard ratios (HRs) with 95% confidence intervals (CIs) were reported or could be calculated with available data according to the approach described by Tierney et al. [18]. We excluded studies if they included less than 50 patients or without sufficient data to calculate HRs and 95% CIs. For overlapped cohorts, only the latest and most intact report was included.

### Data extraction and quality assessment

Two authors independently extracted data from eligible studies using a data collection form including the following items: first author, year of publication, study region, recruitment time, study design, ethnicity, sample size, sex distribution, age range, TKI used, transplant status, number of cases with p190/p210, follow-up time, endpoint, statistical method, and HR with 95% CI.

The Quality in Prognosis Studies (QUIPS) tool [19] was used to assess the risk of bias of the included studies in six dimensions: study participation, study attrition, prognostic factor measurement, outcome measurement, study confounding, statistical analysis and reporting. The risk of bias for each domain was indicated as low, moderate or high according to the rating criteria. The overall risk of bias for individual studies was marked as high if one or more domains were rated as high risk of bias, or moderate if one or more domains were rated as moderate risk of bias, or low if all domains were rated as low risk of bias.

The study screening, data extraction and quality assessment procedure were all conducted by two authors independently. Any discrepancies were resolved by consulting a third author.

### Statistical analyses

The primary endpoints were EFS and OS. DFS and RFS were interpreted as synonymous with EFS. HRs and 95% CIs for EFS and OS were pooled to assess the prognostic value of BCR-ABL1 isoforms. If both univariate and multivariate analyses results were presented, we used the latter. If HRs with 95% CIs were not provided, we estimated the data from Kaplan-Meier survival curves using the methods described by Tierney et al. [18].

The heterogeneity between included studies was evaluated using chi-square based Q-test and *I*^*2*^ test. Random-effects model was applied to pool the HRs and 95% CIs if significant heterogeneity existed (P < 0.10 or *I*^*2*^ > 50%). Otherwise, fixed-effects model was used. Subgroup analyses were further performed to assess the potential effect modification of the factors including study region, study design, ethnicity, age group, type of TKI used and transplant status.

Publication bias was evaluated using Begg's funnel plots and Egger's test, with significance defined as P < 0.05. Sensitivity analyses were performed to evaluate single study's influence on pooled HRs by sequentially omitting one study at a time. All of the analyses were performed with Stata version 16.0 software (Stata Corporation, College Station, TX, USA).

## Results

### Literature selection and study characteristics

**Fig 1:**
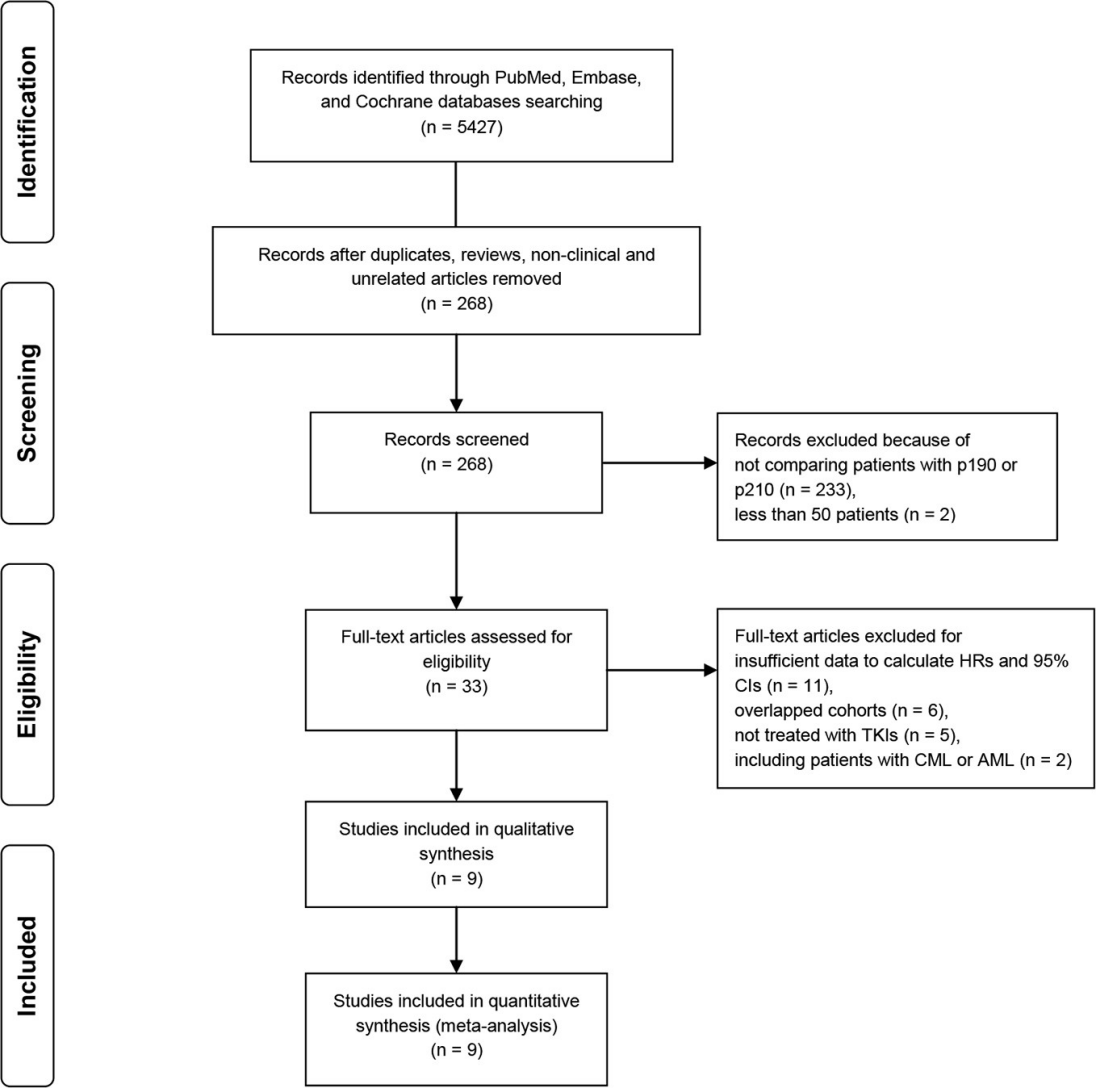
PRISMA flow diagram for study review and inclusion.

Initial literature searches in PubMed, Embase and Cochrane databases identified 5427 articles published till June 15, 2020. After excluding duplicates, reviews, non-clinical and irrelevant studies, 268 articles were deemed to be potentially relevant for further review. A total of 259 studies were excluded for the following reasons: not comparing the clinical outcomes of patients with p190 or p210 (n = 233), insufficient data to calculate HRs and 95% CIs (n = 11), overlapped cohorts (n = 6), patients not or not all treated with TKIs (n = 5), less than 50 patients (n = 2), including patients with chronic myeloid leukemia (CML) or acute myeloid leukemia (AML) (n = 2). Finally, nine studies were included in this meta-analysis [13, 20–27]. The study selection process is presented in Fig 1.

The characteristics of the included nine studies are summarized in Table 1. The raw data is supplied in S1 Data. These studies were published from 2012 onwards with the patient recruitment time ranging from 2001 to 2017, including two prospective cohort studies and seven retrospective cohort studies. The nine studies were all composed of adult patients with de novo Ph+ ALL. The sample size ranged from 57 to 850, with a total number of 1582 Ph+ ALL patients. Two studies were conducted in Europe, five in the East Asia, and two in the USA. In respect to TKIs, four studies used the first-generation TKI imatinib, one study applied the second-generation TKI dasatinib, and four studies recruited patients treated with the first (imatinib)- or second (dasatinib)- or third-generation (ponatinib) TKIs. HSCT was conducted in all patients in two studies, and in a subset of patients in seven studies.

**Table 1:** Characteristics of the studies included in this meta-analysis.

**First author**	**Year**	**Region**	**Time**	**Study design**	**Ethnicity**	**Number (with breakpoint)**	**Male/female**	**Age (years)**
Mizuta S [20]	2012	East Asia	2002.09–2005.05	prospective	Asian	60 (60)	32/28	37 (15–64)
Rousselot P [21]	2016	Europe	2007.08–2010.07	prospective	Caucasian	71 (71)	30/41	69 (59–83)
Qiu LL [13]	2016	East Asia	2005.01–2014.12	retrospective	Asian	65 (65)	38/27	36 (14–66)
DeBoer R [22]	2016	USA	2002.04–2010.04	retrospective	Caucasian	57 (50)	25/32	44 (22–58)
Short NJ [24]	2017	USA	2001.06–2016.01	retrospective	Caucasian	152 (151)	NA	55 (19–85)
Yu GP [23]	2017	East Asia	2010.01–2016.01	retrospective	Asian	77 (77)	52/25	30 (13–59)
Fedullo AL [27]	2019	Europe	NA	retrospective	Caucasian	116 (115)	55/61	51 (19–89)
Huang AJ [25]	2019	East Asia	2007.01–2017.12	retrospective	Asian	134 (134)	71/63	38 (14–60)
Akahoshi Y [26]	2019	East Asia	2001.01–2016.12	retrospective	Asian	850 (832)	483/367	46 (16–71)
**First author**	**Year**	**TKI**	**Transplant status**	**p190/p210**	**Follow-up**	**Endpoint**	**Multi/Univariate**	**HR (95% CI)**
Mizuta S [20]	2012	imatinib	transplant	42/18	31m (12-56m)	DFS	multivariate	3.20 (1.21–8.50)
Rousselot P [21]	2016	dasatinib	mix	54/17	32m (2-88m)	RFS	univariate	1.25 (0.64–2.46)
						OS	univariate	1.32 (0.67–2.60)
Qiu LL [13]	2016	imatinib	mix	41/24	21.6m (0.8–124.7m)	EFS	survival curve	0.76 (0.29–2.00)
						OS	survival curve	1.01 (0.16–6.67)
DeBoer R [22]	2016	imatinib	mix	33/17	6.5yrs (NA)	DFS	survival curve	3.03 (1.22–7.69)
						OS	survival curve	2.56 (0.76–8.33)
Short NJ [24]	2017	imatinib/dasatinib/ponatinib	mix	109/42	43m (2-173m)	RFS	univariate	1.49 (0.82–2.70)
						OS	univariate	1.75 (0.93–3.33)
Yu GP [23]	2017	imatinib/dasatinib	mix	51/26	456d (59-2327d)	DFS	univariate	0.65 (0.24–1.76)
						OS	univariate	0.60 (0.21–1.75)
Fedullo AL [27]	2019	imatinib/dasatinib	mix	70/29	NA	DFS	univariate	1.06 (0.66–1.72)
Huang AJ [25]	2019	imatinib	mix	98/36	32m (4-118m)	DFS	univariate	1.52 (0.83–2.78)
						OS	univariate	1.33 (0.70–2.56)
Akahoshi Y [26]	2019	imatinib/dasatinib	transplant	639/169	1539d (91-5672d)	OS	univariate	0.99 (0.73–1.35)

Abbreviations: NA, not available; TKI, tyrosine kinase inhibitor; d, days; m, months; yrs, years.

According to the QUIPS tool, eight studies were ranked as low risk of bias, and one was moderate risk of bias. The details are listed in Table 2.

**Table 2:** Quality assessment of the included studies using the QUIPS tool.

First author	Year	Study Participation	Study Attrition	Prognostic Factor Measurement	Outcome Measurement	Study Confounding	Statistical Analysis and Reporting	Total
Mizuta S	2012	low	low	low	low	low	low	low
Rousselot P	2016	low	low	low	low	low	low	low
Qiu LL	2016	low	low	low	low	low	low	low
DeBoer R	2016	low	low	low	low	moderate	low	moderate
Short NJ	2017	low	low	low	low	low	low	low
Yu GP	2017	low	low	low	low	low	low	low
Fedullo AL	2019	low	low	low	low	low	low	low
Huang AJ	2019	low	low	low	low	low	low	low
Akahoshi Y	2019	low	low	low	low	low	low	low

### Association between BCR-ABL1 isoforms and EFS

**Fig 2:**
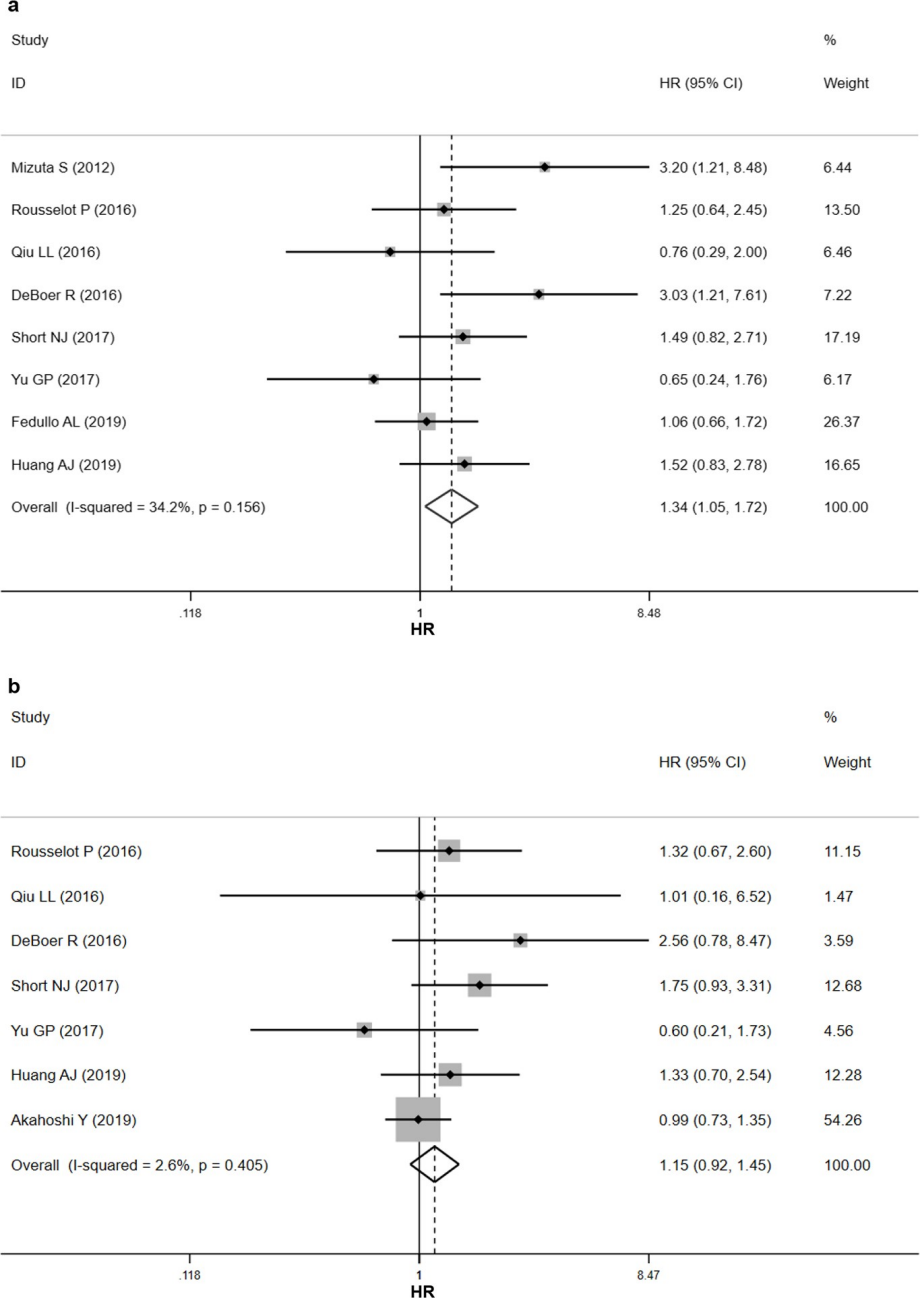
Forest plot for the impact of BCR-ABL1 isoforms on the clinical outcomes of Ph+ ALL patients. (a) Forest plot for event-free survival (EFS). (b) Forest plot for overall survival (OS).

Eight studies investigated the correlation between BCR-ABL1 isoforms and EFS. Pooled HR indicated that p210 was associated with inferior EFS slightly (HR = 1.34, 95% CI 1.05–1.72) (Fig 2A). The effect tended to be adverse in six studies, with the confidence intervals being statistically significant in two of the six studies. In the rest two studies, the effect tended to be favorable, however, with non-significant confidence intervals. No significant heterogeneity existed among the studies (P = 0.156, *I*^*2*^ = 34.2%) and fixed-effects model was employed to pool the HRs. In subgroup analyses, the adverse effect was persistent in a minority of subgroups, including in the study region of USA (HR = 1.84, 95% CI 1.12–3.04), in the ethnicity of Caucasian (HR = 1.36, 95% CI 1.00–1.84), in studies applying the first-generation TKI (HR = 1.75, 95% CI 1.16–2.63), and in the transplant cohort (HR = 3.20, 95% CI 1.21–8.48) (Table 3).

**Table 3:** Subgroup analyses of the impact of BCR-ABL1 isoforms on the prognoses of Ph+ ALL.

	EFS					OS				
	No.	metaHR (95% CI)	P_h_	I^2^ (%)	Effect model	No.	metaHR (95% CI)	P_h_	I^2^ (%)	Effect model
**Overall**	8	1.34 (1.05–1.72)	0.156	34.2	F	7	1.15 (0.92–1.45)	0.405	2.6	F
**Region**										
East Asia	4	1.27 (0.67–2.42)	0.087	54.3	R	4	1.01 (0.77–1.32)	0.647	0.0	F
Europe	2	1.12 (0.76–1.66)	0.703	0.0	F	1	1.32 (0.67–2.60)	/	/	/
USA	2	1.84 (1.12–3.04)	0.206	37.5	F	2	1.91 (1.09–3.34)	0.583	0.0	F
**Study design**										
prospective	2	1.87 (0.75–4.64)	0.120	58.7	R	1	1.32 (0.67–2.60)	/	/	/
retrospective	6	1.27 (0.96–1.67)	0.194	32.2	F	6	1.13 (0.89–1.44)	0.307	16.5	F
**Ethnicity**										
Caucasian	4	1.36 (1.00–1.84)	0.255	26.1	F	3	1.64 (1.07–2.53)	0.614	0.0	F
Asian	4	1.27 (0.67–2.42)	0.087	54.3	R	4	1.01 (0.77–1.32)	0.647	0.0	F
**Age group**										
15–39	4	1.27 (0.67–2.42)	0.087	54.3	R	3	1.07 (0.63–1.81)	0.451	0.0	F
40–59	3	1.38 (0.98–1.96)	0.136	49.9	F	3	1.38 (0.81–2.36)	0.114	53.9	R
≥ 60	1	1.25 (0.64–2.45)	/	/	/	1	1.32 (0.67–2.60)	/	/	/
**TKI**										
1st-generation	4	1.75 (1.16–2.63)	0.117	49.2	F	3	1.49 (0.87–2.57)	0.585	0.0	F
2nd-generation	1	1.25 (0.64–2.45)	/	/	/	1	1.32 (0.67–2.60)	/	/	/
mix	3	1.13 (0.79–1.60)	0.354	3.8	F	3	1.06 (0.81–1.39)	0.157	46.1	F
**Cohort**										
transplant	1	3.20 (1.21–8.48)	/	/	/	1	0.99 (0.73–1.35)	/	/	/
mix	6	1.27 (0.98–1.63)	0.288	18.6	F	6	1.38 (0.99–1.93)	0.537	0.0	F

Abbreviations: P_h_, P value for heterogeneity; F, fixed-effects model; R, random-effects model.

### Association between BCR-ABL1 isoforms and OS

Seven studies explored the influence of BCR-ABL1 isoforms on OS. No significant heterogeneity exited across studies (P = 0.405, *I*^*2*^ = 2.6%) and fixed-effects model was applied. Combined HR suggested that BCR-ABL1 isoforms did not significantly affect the OS of patients with Ph+ ALL (HR = 1.15, 95% CI 0.92–1.45) (Fig 2B). The confidence intervals crossed the null value in all the seven studies, although a trend toward adverse effect was seen in five studies. In subgroup analyses, the pooled HRs were only statistically significant in the study region of USA (HR = 1.91, 95% CI 1.09–3.34) and in the ethnicity of Caucasian (HR = 1.64, 95% CI 1.07–2.53) (Table 3).

### Publication bias and sensitivity analyses

**Fig 3:**
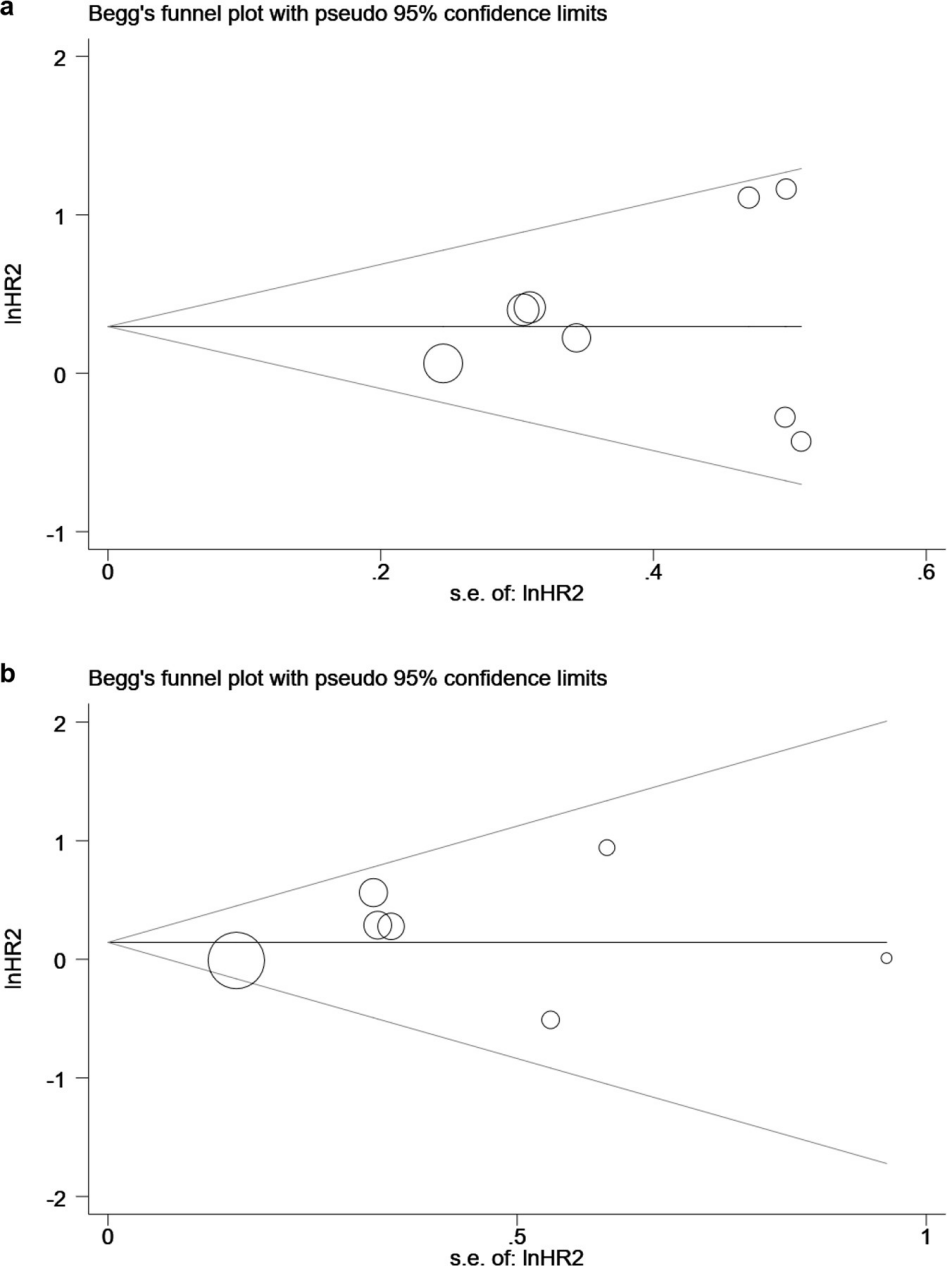
Begg's test of publication bias for (a) event-free survival (EFS) and (b) overall survival (OS).

**Fig 4:**
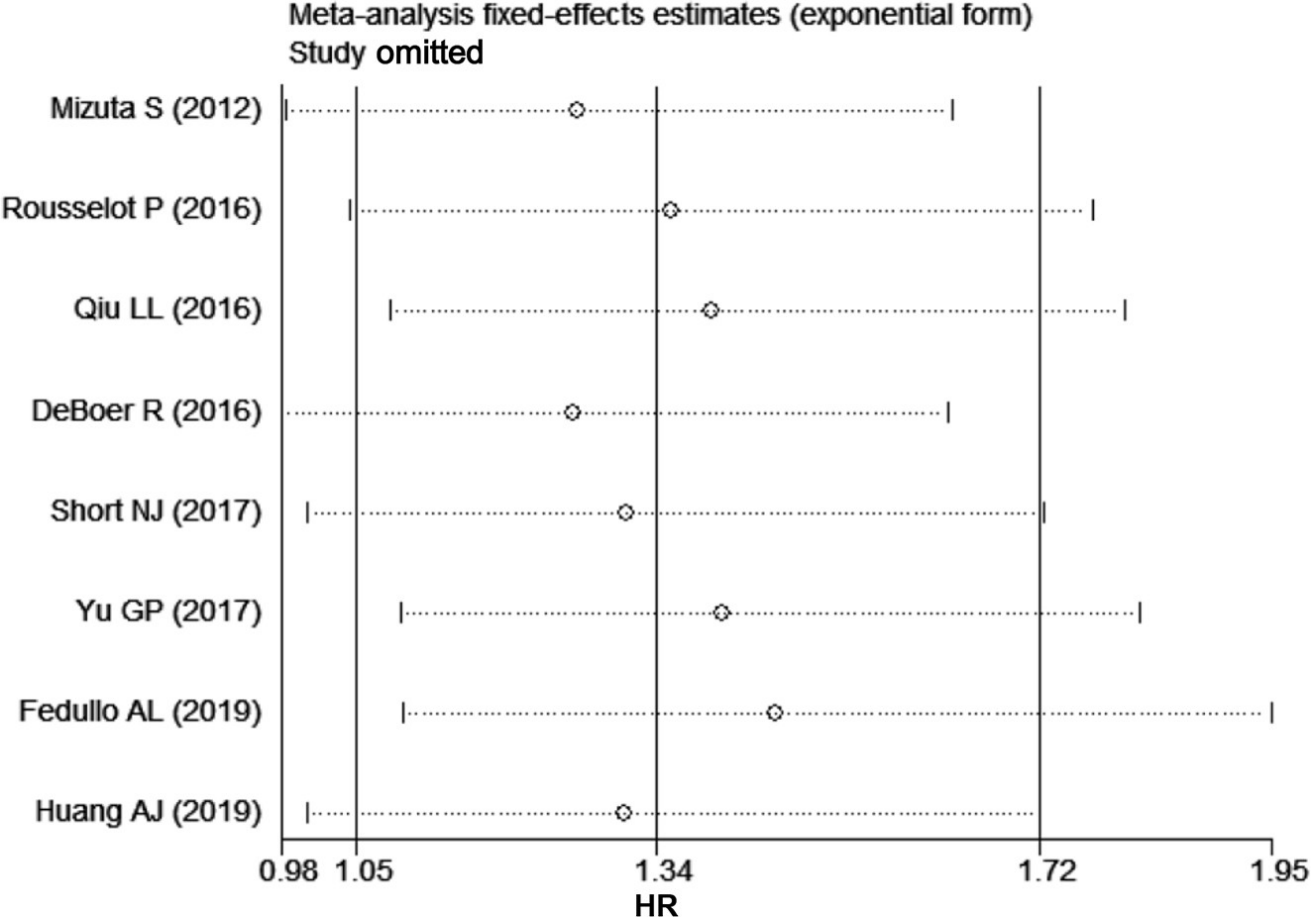
Sensitivity analysis for event-free survival (EFS).

Begg's or Egger's test showed no publication bias for EFS (P = 1.000 or 0.641, respectively) or OS (P = 1.000 or 0.452, respectively) (Fig 3). Sensitivity analyses revealed that no single study significantly altered the results (Fig 4).

## Discussion

Despite the tremendous outcome improvement for Ph+ ALL patients in the TKI era, resistance or relapse still remains a problem yet to be resolved for clinicians. The heterogeneous clinical outcomes suggest the necessity of identifying potential high risk factors so as to adopt more individualized therapeutic regimens. BCR-ABL1 fusion gene is the primary oncogenic driver mutation of Ph+ ALL. Two different transcripts (p190 or p210), resulting from different fusion patterns of the two genes, are detected in most Ph+ ALL patients. Whether the BCR-ABL1 isoforms imply different clinical outcomes is still controversial in previous studies. Thus, we conducted herein a meta-analysis to clarify the prognostic value of BCR-ABL1 isoforms in Ph+ ALL patients.

The present meta-analysis included nine studies with a total number of 1582 Ph+ ALL patients. Combined HRs suggested that p210 posed a mild adverse impact on EFS yet not OS. The biological and clinical heterogeneity of BCR-ABL1 isoforms might account for the diverse influence on clinical outcomes. Nagel et al. found that patients with p210 mainly showed hematopoietic stem cell involvement, whereas in most patients with p190, only the CD19+ leukemia compartments were BCR-ABL1 positive [28]. As to disease presenting features, patients with p190 were described with younger age [4] and lower presenting white blood cell count [29]. In the study of Chiaretti et al., treated with imatinib plus chemotherapy, patients with p190 showed a significantly faster molecular response than patients with p210 [30]. Yu et al. also showed a higher major molecular response rate after the first cycle of induction therapy in patients with p190 than in patients with p210 [23]. In contrast to Ph+ ALL, however, the presence of p190 is associated with higher risk of disease progression and inferior cytogenetic and molecular responses to TKI therapy in patients with CML [31–33].

In subgroup analysis of EFS, the effect was statistically significant in the subgroup of first-generation TKI (imatinib) treated cohorts, yet not in the next generation TKIs treated cohorts, suggesting that the adverse impact of p210 might be overcome by next generation TKIs. This suggested the possibility that second- or third-generation TKIs might be a preferred choice for patients with p210. In subgroup analysis of transplant status, the adverse effect was statistically significant in the transplant cohort, while not in the studies recruiting both transplant or non-transplant patients. It should be noted that the transplant subgroup included only one study. Further studies are needed to validate the result.

The heterogeneity in the treatment responses and clinical outcomes of patients with Ph+ ALL has aroused particular interest in the investigation of additional genomic lesions and potential prognostic markers. Through genome-wide analysis, researchers found that the most commonly detected alterations were those targeting the IKZF1, PAX5 and CDKN2A/B genes, with deletions being the dominant anomaly patterns [34, 35]. IKZF1 or CDKN2A/B deletions were demonstrated to be adverse prognostic factors for Ph+ ALL patients in several studies [36–39], and there were evidences that these might not be overcome by allo-HSCT [35, 40]. Fedullo et al. once investigated the copy number aberrations (CNA) in 116 de novo Ph+ ALL patients and revealed that simultaneous deletions of IKZF1 plus CDKN2A/B and/or PAX5 conferred a significantly worse DFS [27]. We have performed two meta-analyses to assess the prognostic value of IKZF1 and CDKN2A/B deletions in patients with ALL [41, 42]. In the Ph+ ALL subgroup, the adverse impact on EFS and OS was statistically significant for CDKN2A/B deletions but not for IKZF1 deletions. Minimal residual disease (MRD) was another extensively investigated prognostic factor. In the systematic review and meta-analysis performed by Bassan et al., MRD positivity was associated with inferior RFS and OS in the Ph+ ALL subgroup [43]. A refined risk stratification algorithm comprising disease presenting features, additional genomic lesions, MRD and mutations at relapse need to be proposed to help inform disease prognosis and optimize treatment strategies for individual patients.

Several limitations should be considered in this meta-analysis. Firstly, due to the rarity of p230, we could not achieve enough data to analyze its impact on prognosis. Secondly, some of the HRs were extracted from Kaplan-Meier curves, which might be less reliable than the original data or multivariate analysis results. Thirdly, specific BCR-ABL1 kinase domain mutations might be associated with BCR-ABL1 isoforms. However, due to few data available in the included studies, this was not considered in our analysis.

In conclusion, our meta-analysis showed that p210 was slightly associated with inferior EFS of patients with Ph+ ALL. The negative effect might be overcome by second- or third-generation TKIs. Further prospective studies are needed to verify our findings.

## Supporting information

S1 Checklist PRISMA 2009 checklist. (DOC)

S1 File Search strategy for Pubmed. (DOCX)

S1 Data Raw data of this meta-analysis. (XLSX)

## References

[1] Burmeister T, Schwartz S, Bartram CR, Gökbuget N, Hoelzer D, Thiel E. Patients' age and BCR-ABL frequency in adult B-precursor ALL: a retrospective analysis from the GMALL study group. Blood. 2008; 112(3): 918–919. 10.1182/blood-2008-04-149286 18650471

[2] Moorman AV, Chilton L, Wilkinson J, Ensor HM, Bown N, Proctor SJ. A population-based cytogenetic study of adults with acute lymphoblastic leukemia. Blood. 2010; 115(2): 206–214. 10.1182/blood-2009-07-232124 19897583

[3] Dombret H, Gabert J, Boiron JM, Rigal-Huguet F, Blaise D, Thomas X, et al Outcome of treatment in adults with Philadelphia chromosome-positive acute lymphoblastic leukemia—results of the prospective multicenter LALA-94 trial. Blood. 2002; 100(7): 2357–2366. 10.1182/blood-2002-03-0704 12239143

[4] Gleissner B, Gökbuget N, Bartram CR, Janssen B, Rieder H, Janssen JW, et al Leading prognostic relevance of the BCR-ABL translocation in adult acute B-lineage lymphoblastic leukemia: a prospective study of the German Multicenter Trial Group and confirmed polymerase chain reaction analysis. Blood. 2002; 99(5): 1536–1543. 10.1182/blood.v99.5.1536 11861265

[5] Fielding AK, Rowe JM, Richards SM, Buck G, Moorman AV, Durrant IJ, et al Prospective outcome data on 267 unselected adult patients with Philadelphia chromosome-positive acute lymphoblastic leukemia confirms superiority of allogeneic transplantation over chemotherapy in the pre-imatinib era: results from the International ALL Trial MRC UKALLXII/ECOG2993. Blood. 2009; 113(19): 4489–4496. 10.1182/blood-2009-01-199380 19244158 PMC4188540

[6] Daver N, Thomas D, Ravandi F, Cortes J, Garris R, Jabbour E, et al Final report of a phase II study of imatinib mesylate with hyper-CVAD for the front-line treatment of adult patients with Philadelphia chromosome-positive acute lymphoblastic leukemia. Haematologica. 2015; 100(5): 653–661. 10.3324/haematol.2014.118588 25682595 PMC4420214

[7] Lim SN, Joo YD, Lee KH, Kim DY, Lee JH, Lee JH, et al Long-term follow-up of imatinib plus combination chemotherapy in patients with newly diagnosed Philadelphia chromosome-positive acute lymphoblastic leukemia. Am J Hematol. 2015; 90(11): 1013–1020. 10.1002/ajh.24137 26228525

[8] Ravandi F, O'Brien SM, Cortes JE, Thomas DM, Garris R, Faderl S, et al Long-term follow-up of a phase 2 study of chemotherapy plus dasatinib for the initial treatment of patients with Philadelphia chromosome-positive acute lymphoblastic leukemia. Cancer. 2015; 121(23): 4158–4164. 10.1002/cncr.29646 26308885 PMC4666803

[9] Kim DY, Joo YD, Lim SN, Kim SD, Lee JH, Lee JH, et al Nilotinib combined with multiagent chemotherapy for newly diagnosed Philadelphia-positive acute lymphoblastic leukemia. Blood. 2015; 126(6): 746–756. 10.1182/blood-2015-03-636548 26065651

[10] Jabbour E, Kantarjian H, Ravandi F, Thomas D, Huang X, Faderl S, et al Combination of hyper-CVAD with ponatinib as first-line therapy for patients with Philadelphia chromosome-positive acute lymphoblastic leukaemia: a single-centre, phase 2 study. Lancet Oncol. 2015; 16(15): 1547–1555. 10.1016/S1470-2045(15)00207-7 26432046 PMC4816046

[11] de Klein A, van Kessel AG, Grosveld G, Bartram CR, Hagemeijer A, Bootsma D, et al A cellular oncogene is translocated to the Philadelphia chromosome in chronic myelocytic leukaemia. Nature. 1982; 300(5894): 765–767. 10.1038/300765a0 6960256

[12] Bernt KM, Hunger SP. Current concepts in pediatric Philadelphia chromosome-positive acute lymphoblastic leukemia. Front Oncol. 2014; 4: 54 10.3389/fonc.2014.00054 24724051 PMC3971203

[13] Qiu LL, Lu YJ, Jing Y, Yu L, Liu DH, Wang LL. [Comparison of Clinical Outcomes between P190 and P210 Trans-cripts in Adult Ph Chromosome Positive Acute Lymphoblastic Leukemia in the New Era of TKI]. Zhongguo Shi Yan Xue Ye Xue Za Zhi. 2016; 24(2): 369–374. 10.7534/j.issn.1009-2137.2016.02.012 27150994

[14] Lugo TG, Witte ON. The BCR-ABL oncogene transforms Rat-1 cells and cooperates with v-myc. Mol Cell Biol. 1989; 9(3): 1263–1270. 10.1128/mcb.9.3.1263 2725497 PMC362717

[15] Kelliher M, Knott A, McLaughlin J, Witte ON, Rosenberg N. Differences in oncogenic potency but not target cell specificity distinguish the two forms of the BCR/ABL oncogene. Mol Cell Biol. 1991; 11(9): 4710–4716. 10.1128/mcb.11.9.4710 1875948 PMC361365

[16] Voncken JW, Kaartinen V, Pattengale PK, Germeraad WT, Groffen J, Heisterkamp N. BCR/ABL P210 and P190 cause distinct leukemia in transgenic mice. Blood. 1995; 86(12): 4603–4611. 8541551

[17] Moher D, Liberati A, Tetzlaff J, Altman DG. Preferred reporting items for systematic reviews and meta-analyses: the PRISMA statement. J Clin Epidemiol. 2009; 62(10): 1006–1012. 10.1016/j.jclinepi.2009.06.005 19631508

[18] Tierney JF, Stewart LA, Ghersi D, Burdett S, Sydes MR. Practical methods for incorporating summary time-to-event data into meta-analysis. Trials. 2007; 8: 16 10.1186/1745-6215-8-16 17555582 PMC1920534

[19] Hayden JA, van der Windt DA, Cartwright JL, Côté P, Bombardier C. Assessing bias in studies of prognostic factors. Ann Intern Med. 2013; 158(4): 280–286. 10.7326/0003-4819-158-4-201302190-00009 23420236

[20] Mizuta S, Matsuo K, Maeda T, Yujiri T, Hatta Y, Kimura Y, et al Prognostic factors influencing clinical outcome of allogeneic hematopoietic stem cell transplantation following imatinib-based therapy in BCR-ABL-positive ALL. Blood Cancer J. 2012; 2(5): e72 10.1038/bcj.2012.18 22829974 PMC3366071

[21] Rousselot P, Coudé MM, Gokbuget N, Gambacorti Passerini C, Hayette S, Cayuela JM, et al Dasatinib and low-intensity chemotherapy in elderly patients with Philadelphia chromosome-positive ALL. Blood. 2016; 128(6): 774–782. 10.1182/blood-2016-02-700153 27121472 PMC5085255

[22] DeBoer R, Koval G, Mulkey F, Wetzler M, Devine S, Marcucci G, et al Clinical impact of ABL1 kinase domain mutations and IKZF1 deletion in adults under age 60 with Philadelphia chromosome-positive (Ph+) acute lymphoblastic leukemia (ALL): molecular analysis of CALGB (Alliance) 10001 and 9665. Leuk Lymphoma. 2016; 57(10): 2298–2306. 10.3109/10428194.2016.1144881 26892479 PMC5008253

[23] Yu G, Chen F, Yin C, Liu Q, Sun J, Xuan L, et al Upfront treatment with the first and second-generation tyrosine kinase inhibitors in Ph-positive acute lymphoblastic leukemia. Oncotarget. 2017; 8(63): 107022–107032. 10.18632/oncotarget.22206 29291008 PMC5739793

[24] Short NJ, Kantarjian HM, Sasaki K, Ravandi F, Ko H, Cameron Yin C, et al Poor outcomes associated with +der(22)t(9;22) and -9/9p in patients with Philadelphia chromosome-positive acute lymphoblastic leukemia receiving chemotherapy plus a tyrosine kinase inhibitor. Am J Hematol. 2017; 92(3): 238–243. 10.1002/ajh.24625 28006851 PMC5495018

[25] Huang AJ, Wang LB, Du J, Tang GS, Cheng H, Gong SL, et al [Efficacy of Hyper-CVAD/MA and CHALL-01 regimens in the treatment of Philadelphia chromosome-positive adult acute lymphoblastic leukemia patients under 60 years old]. Zhonghua Xue Ye Xue Za Zhi. 2019; 40(8): 625–632. 10.3760/cma.j.issn.0253-2727.2019.08.001 31495127 PMC7342869

[26] Akahoshi Y, Nishiwaki S, Mizuta S, Ohashi K, Uchida N, Tanaka M, et al Tyrosine kinase inhibitor prophylaxis after transplant for Philadelphia chromosome-positive acute lymphoblastic leukemia. Cancer Sci. 2019; 110(10): 3255–3266. 10.1111/cas.14167 31402561 PMC6778639

[27] Fedullo AL, Messina M, Elia L, Piciocchi A, Gianfelici V, Lauretti A, et al Prognostic implications of additional genomic lesions in adult Philadelphia chromosome-positive acute lymphoblastic leukemia. Haematologica. 2019; 104(2): 312–318. 10.3324/haematol.2018.196055 30190342 PMC6355475

[28] Nagel I, Bartels M, Duell J, Oberg HH, Ussat S, Bruckmueller H. Hematopoietic stem cell involvement in BCR-ABL1-positive ALL as a potential mechanism of resistance to blinatumomab therapy. Blood. 2017; 130(18): 2027–2031. 10.1182/blood-2017-05-782888 28827408 PMC5726343

[29] Cimino G, Pane F, Elia L, Finolezzi E, Fazi P, Annino L, et al The role of BCR/ABL isoforms in the presentation and outcome of patients with Philadelphia-positive acute lymphoblastic leukemia: a seven-year update of the GIMEMA 0496 trial. Haematologica. 2006; 91(3): 377–380. 16531262

[30] Chiaretti S, Vitale A, Vignetti M, Piciocchi A, Fazi P, Elia L, et al A sequential approach with imatinib, chemotherapy and transplant for adult Ph+ acute lymphoblastic leukemia: final results of the GIMEMA LAL 0904 study. Haematologica. 2016; 101(12): 1544–1552. 10.3324/haematol.2016.144535 27515250 PMC5479612

[31] Gong Z, Medeiros LJ, Cortes JE, Zheng L, Khoury JD, Wang W, et al Clinical and prognostic significance of e1a2 BCR-ABL1 transcript subtype in chronic myeloid leukemia. Blood Cancer J. 2017; 7(7): e583 10.1038/bcj.2017.62 28708130 PMC5549254

[32] Qin YZ, Jiang Q. Prevalence and outcomes of uncommon BCR-ABL1 fusion transcripts in patients with chronic myeloid leukaemia: data from a single centre. Br J Haematol. 2018; 182(5): 693–700. 10.1111/bjh.15453 29974949

[33] Xue M, Wang Q, Huo L, Wen L, Yang X, Wu Q. Clinical characteristics and prognostic significance of chronic myeloid leukemia with rare BCR-ABL1 transcripts. Leuk Lymphoma. 2019; 60(12): 3051–3057. 10.1080/10428194.2019.1607329 31258010

[34] Mullighan CG, Miller CB, Radtke I, Phillips LA, Dalton J, Ma J, et al BCR-ABL1 lymphoblastic leukaemia is characterized by the deletion of Ikaros. Nature. 2008; 453(7191): 110–114. 10.1038/nature06866 18408710

[35] Pfeifer H, Raum K, Markovic S, Nowak V, Fey S, Obländer J, et al Genomic CDKN2A/2B deletions in adult Ph(+) ALL are adverse despite allogeneic stem cell transplantation. Blood. 2018; 131(13): 1464–1475. 10.1182/blood-2017-07-796862 29348129

[36] Martinelli G, Iacobucci I, Storlazzi CT, Vignetti M, Paoloni F, Cilloni D, et al IKZF1 (Ikaros) deletions in BCR-ABL1-positive acute lymphoblastic leukemia are associated with short disease-free survival and high rate of cumulative incidence of relapse: a GIMEMA AL WP report. J Clin Oncol. 2009; 27(31): 5202–5207. 10.1200/JCO.2008.21.6408 19770381

[37] Li H, Zhang W, Kuang P, Ye Y, Yang J, Dai Y, et al Combination of IKZF1 deletion and early molecular response show significant roles on prognostic stratification in Philadelphia chromosome-positive acute lymphoblastic leukemia patients. Leuk Lymphoma. 2018; 59(8): 1890–1898. 10.1080/10428194.2017.1406933 29214878

[38] Iacobucci I, Ferrari A, Lonetti A, Papayannidis C, Paoloni F, Trino S, et al CDKN2A/B alterations impair prognosis in adult BCR-ABL1-positive acute lymphoblastic leukemia patients. Clin Cancer Res. 2011; 17(23): 7413–7423. 10.1158/1078-0432.CCR-11-1227 22134481

[39] Xu N, Li YL, Li X, Zhou X, Cao R, Li H, et al Correlation between deletion of the CDKN2 gene and tyrosine kinase inhibitor resistance in adult Philadelphia chromosome-positive acute lymphoblastic leukemia. J Hematol Oncol. 2016; 9: 40 10.1186/s13045-016-0270-5 27090891 PMC4836197

[40] Kim M, Park J, Kim DW, Kim YJ, Jeon YW, Yoon JH, et al Impact of IKZF1 deletions on long-term outcomes of allo-SCT following imatinib-based chemotherapy in adult Philadelphia chromosome-positive ALL. Bone Marrow Transplant. 2015; 50(3): 354–362. 10.1038/bmt.2014.281 25501350

[41] Zhang W, Kuang P, Li H, Wang F, Wang Y. Prognostic significance of IKZF1 deletion in adult B cell acute lymphoblastic leukemia: a meta-analysis. Ann Hematol. 2017; 96(2): 215–225. 10.1007/s00277-016-2869-6 27815723

[42] Zhang W, Kuang P, Liu T. Prognostic significance of CDKN2A/B deletions in acute lymphoblastic leukaemia: a meta-analysis. Ann Med. 2019; 51(1): 28–40. 10.1080/07853890.2018.1564359 30592434 PMC7857473

[43] Bassan R, Brüggemann M, Radcliffe HS, Hartfield E, Kreuzbauer G, Wetten S. A systematic literature review and meta-analysis of minimal residual disease as a prognostic indicator in adult B-cell acute lymphoblastic leukemia. Haematologica. 2019; 104(10): 2028–2039. 10.3324/haematol.2018.201053 30890593 PMC6886415

