# Supplementary material for: Role of BCR-ABL1 isoforms on the prognosis of Philadelphia chromosome positive acute lymphoblastic leukemia in the tyrosine kinase inhibitor era: A meta-analysis
Source: PLoS One. 2020 Dec 18;15(12):e0243657. doi: 10.1371/journal.pone.0243657 (PMC7748129; doi:10.1371/journal.pone.0243657)
Supplement: S1 File — (DOCX) [file pone.0243657.s002.docx]

Pubmed

Search ((((("Philadelphia Chromosome"[Mesh]) OR BCR-ABL1)) AND "Precursor Cell Lymphoblastic Leukemia-Lymphoma"[Mesh])) OR ((Ph positive acute lymphoblastic leukemia) OR BCR-ABL1 positive acute lymphoblastic leukemia)
